# The Catalytic Role of RuBisCO for *in situ* CO_2_ Recycling in *Escherichia coli*

**DOI:** 10.3389/fbioe.2020.543807

**Published:** 2020-11-30

**Authors:** Ju-Jiun Pang, Jong-Shik Shin, Si-Yu Li

**Affiliations:** ^1^Department of Chemical Engineering, National Chung Hsing University, Taichung City, Taiwan; ^2^Department of Biotechnology, Yonsei University, Seoul, South Korea; ^3^Innovation and Development Center of Sustainable Agriculture, National Chung Hsing University, Taichung City, Taiwan

**Keywords:** Carbon dioxide, mixotroph, Ribulose-1, 5-bisphosphate carboxylase/oxygenase, RuBisCo activase, *Acidithiobacillus ferrooxidans*

## Abstract

Ribulose-1,5-bisphosphate carboxylase/oxygenase (RuBisCO) is a key enzyme responsible for biological CO_2_ assimilation. RuBisCO can be heterologously expressed in *Escherichia coli* so that glucose and CO_2_ are co-metabolized to achieve high mixotrophic metabolite production, where the theoretical yield of mixotrophic metabolite production is 2.4 mol_(ethanol__+__acetate__+__pyruvate)_/mol_glucose_. However, RuBisCO is known for its low k_cat_ and for forming inhibited complexes with its substrate ribulose-1,5-bisphosphate (RuBP) and other sugar phosphates, yet the inhibited form of RuBisCO can be reversed by RuBisCO activase (Rca). In this study, RuBisCO forms I and II were cloned and expressed in *Escherichia coli* for *in situ* CO_2_ recycling, where CO_2_ produced during glucose fermentation was recycled and co-metabolized with the glucose. In addition, forms I and II RuBisCO activases were co-expressed with RuBisCO in *E. coli* to determine their *in vivo* effects on *in situ* CO_2_ recycling. Form I RuBisCO activase (Rca1) was co-expressed with form I RuBisCO and form II RuBisCO activase (Rca2) was co-expressed with form II RuBisCO. The results showed that both form I and form II RuBisCO exhibit comparable activities in *E. coli* and generated similar levels of *in situ* CO_2_ recycling. A significant increase in the total metabolite yield from 1.5 ± 0.1 to 2.2 ± 0.1 mol_(ethanol__+__acetate__+__pyruvate)_/mol_glucose_ occurred when Rca2 was co-expressed with form II RuBisCO. Meanwhile, the total metabolite yield increased from 1.7 ± 0.1 to 2.0 ± 0.1 mol_(ethanol__+__acetate__+__pyruvate)_/mol_glucose_ when Rca1 was co-expressed with form I RuBisCO. This data suggests that both forms I and II RuBisCO are subject to *in vivo* RuBP inhibition yet can be relieved by the co-expression of Rca. Interestingly, it is suggested that the *in vivo* RuBP inhibition of form II RuBisCO can be more easily reversed compared to form I. When the catalytic power of RuBisCO is maintained by Rca, the high activity of phosphoribulokinase (Prk) plays an important role in directing glucose to the RuBisCO-based engineered pathway and fermentation yields of 2.1–2.3 mol_(ethanol__+__acetate__+__pyruvate)_/mol_glucose_ can be obtained. This study is the first to demonstrate that *in vivo* RuBP inhibition of RuBisCO can be a bottleneck for *in situ* CO_2_ recycling in *E. coli*.

## Introduction

The Calvin-Benson-Bassham (CBB) cycle is one of the main metabolic pathways for converting inorganic CO_2_ to organic carbon during photosynthesis (Tabita et al., 2008; Nielsen et al., 2012; François et al., 2020). Ribulose-1, 5-bisphosphate carboxylase/oxygenase (RuBisCO) is a key enzyme of the CBB cycle and is responsible for CO_2_ fixation by catalyzing the carboxylation of RuBP. RuBisCO can be categorized into four groups: form I, II, III, and IV (Badger and Bek, 2008). These four different forms are structurally unique (Tabita et al., 2008). However, to date, only forms I and II RuBisCO have proven to operate in the CBB cycle (Tabita et al., 2008). The first RuBisCO structure determined by X-ray crystallography was from the bacterium *Rhodospirillum rubrum*, which is now categorized as form II (Tabita et al., 2008). This enzyme is composed of only two large subunits (L_2_) which share 25–30% identity to the L subunits of form I RuBisCO. Form II RuBisCO is considered to be an original form of RuBisCO (Tabita et al., 2008), and the catalytic site is at the interface between the L subunits. Conversely, form I RuBisCO is composed of eight L subunits (∼53 kDa) and eight small (S) subunits (∼15 kDa). The basic structural motif, dimer of L, is repeated four times to form a catalytic (L_8_) core with eight S subunits capping the top and the bottom of this core (Tabita et al., 2008). Similarly, the catalytic site of form I RuBisCO is also at the interface between the L subunits. In addition, based on phylogenetic analysis of the amino acid sequence, form I RuBisCO can be further classified as red-like and green-like RuBisCO. Form I RuBisCO is the most abundant enzyme on Earth and can be found in eukaryotes, bacteria, and higher plants (Ashida et al., 2008).

The catalytic activity of RuBisCO requires an activation step. First, non-substrate CO_2_ binds to an active site to convert the conserved Lys^201^ to a carbamylated form, which is then stabilized by Mg^2+^. As a result, the activated form of RuBisCO is capable of converting substrate RuBP and CO_2_ into two molecules of 3-phosphoglycerate (PGA). In contrast, the binding of RuBP to a decarbamylated enzyme prior to CO_2_ leads to a dead-end complex in which activation requires an energy-consuming conformational change of RuBisCO to release the bound RuBP (Parry et al., 2008). RuBisCO is prone to forming inactive complexes; therefore, the activation of RuBisCO is critical for catalysis (McNevin et al., 2006). RuBisCO is also known to be inhibited by other sugar phosphates, such as xylulose-1,5-bisphosphate and 2,3-pentodiulose-1,5-bisphosphate (Parry et al., 2008).

RuBisCO activase (Rca) is known to mediate the release of the inhibitory sugar phosphate from the inactive RuBisCO. It was identified as a biochemical lesion responsible for the high CO_2_-requiring phenotype of the Rca mutant of *Arabidopsis* (Somerville et al., 1982), which lacks the Rca protein (Salvucci et al., 1985). A previous study showed that RuBisCO cannot achieve and maintain an adequate level of activity for growth at ambient CO_2_ without the aid of Rca (McNevin et al., 2006). Rca reactivates RuBisCO by consuming ATP and therefore belongs to the AAA+ superfamily (ATPases associated with diverse cellular activities). Three classes of Rca have been characterized (Mueller-Cajar, 2017). The first class acts on plants whereas the second class (known as CbbX) acts on bacterial red-type form I RuBisCO. In a recent study, a third class of Rca (named CbbQO type Rca) found in a chemolithoautotroph *Acidithiobacillus ferrooxidans* (ATCC 23270) was biochemically characterized (Tsai et al., 2015). Two isoforms of CbbQO protein complexes, form I Rca (Rca1) and form II Rca (Rca2), *in vitro* elevate the activities of forms I and II RuBisCO, respectively, where CbbQ acts as a motor to catalyze ATP hydrolysis and CbbO acts as a functional binder to interact with RuBisCO (Tsai et al., 2015). The native quaternary structure of Rca (Q_6_O_1_) has been classified as a MoxR AAA+ ATPase, where CbbQs form a hexameric ring-shaped conformation. A recent structural and biochemical study has been shown that the axial pore of the hexameric CbbQ ring is critical for CbbQ docking and consequently executing ATPase activity (Tsai et al., 2020). The side of the hexameric CbbQ ring is essential for RuBisCO binding and docking (Tsai et al., 2020).

The partial Calvin cycle was constructed in *Escherichia coli* where phosphoribulokinase (Prk) and RuBisCO (originating from *Synechococcus* PCC6301) were heterologously expressed for directed evolution of RuBisCO (Parikh et al., 2006). Then, the partial Calvin cycle in *E. coli* was investigated for *in situ* CO_2_ recycling during the fermentation of pentoses (Zhuang and Li, 201) and hexose (Li et al., 2015; Yang et al., 2016). Recently, a study showed that this RuBisCO-based engineered *E. coli* [*E. coli* MZLFB (BL21(DE3) *Δzwf Δldh Δfrd*, harboring three genes (*rbcL*, *rbcS*, and *prk*) surpassed the conventional yield of 2 mol_(ethanol__+__acetate__+__pyruvate)_/mol_glucose_ to reach 2.2 mol_(ethanol__+__acetate__+__pyruvate)_/mol_glucose_ (Tseng et al., 2018). This mixotrophic fermentation, where sugar and CO_2_ are simultaneously assimilated for fermentation production, leads to a theoretical yield of fermentation products of 2.4 mol_(ethanol__+__acetate__+__pyruvate)_/mol_glucose_.

Although functional expression of RuBisCO in *E. coli* (Parikh et al., 2006; Tsai et al., 2015; Antonovsky et al., 2016; Tseng et al., 2018) and yeast (Gassler et al., 2019) have been reported, the low k_cat_ of RuBisCO raises the question of how the catalytic performance of RuBisCO affects the rate of CO_2_ fixation. In this study, the effect of forms I and II RuBisCOs from *A. ferrooxidans* (ATCC 23270) on the rate of *in situ* CO_2_ recycling in the RuBisCO-based engineered *E. coli* was investigated. Moreover, the third and newly disclosed class of Rca, RcaQ1O1 (encoded by *cbbQ1* and *cbbO1*, designated as Rca1 in this study) and RcaQ2O2 (encoded by *cbbQ2* and *cbbO2*, designated as Rca2 in this study), from *A. ferrooxidans* (ATCC 23270) (Tsai et al., 2015), were cloned and expressed in RuBisCO-based engineered *E. coli*. The *in vivo* effects of Rca on the regulation of RuBisCO activity were investigated for the first time by monitoring its rate of in *in situ* CO_2_ recycling. The conjugation of form I RuBisCO-Rca1 or form II RuBisCO-Rca2, which are all from *A. ferrooxidans*, was tested in the RuBisCO-based engineered *E. coli*. To demonstrate the generality of this approach, the *in vivo* interaction of Rca1 with form I RuBisCO from a different organism, *Synechococcus* PCC6301 (rbcLS), was also investigated. RbcLS lacks the HK/R motif at the *C*-terminus of the large subunit, where the HK/R motif of form I RuBisCO from *A. ferrooxidans* (AfcbbLS) was proposed to be important for the *in vitro* interaction of AfcbbLS and Rca1 (Tsai et al., 2015). The role of the HK/R motif in the *in vivo* interaction between rbcLS and Rca1 was investigated.

## Materials and Methods

### Bacterial Strains and Plasmids

All strains and plasmids used in this study are listed in Table 1.

**TABLE 1:** List of bacteria strains and plasmids used in this study.

**Name**	**Description**	**Ref.**
**Bacterial strains**		
*E. coli* BL21 (DE3)	F^–^, *dcm*, *ompT*, *gal*, *lon*, *hsd*S_B_(rB^–^, mB^–^), λ(DE3[*lac*I, *lac*UV5-T7 gene 1, *ind*1, *sam*7,*nin*5])	Lab stock
*E. coli* DH5α	F^–^ *endA1 glnV44 thi-1 recA1 relA1 gyrA96 deoR nupG purB20* φ80d*lacZ*ΔM15 Δ(*lacZYA-argF*)U169, hsdR17(*r*_*K*_^–^*m*_*K*_^+^), λ^–^	Lab stock
*E. coli* MZLF	*E. coli* BL21(DE3) *Δzwf* ::FRT *ldh* ::FRT *frd*::FRT	Yang et al., 2016
*E. coli* MZLFA	MZLF harboring pBAD-his6-*prkA*-pACYC184 and rbcLS-pET30a(+)	This study
**Plasmids**		
pBAD-his6-*prkA*-pACYC184	Recombinant plasmid carries *prkA* gene (derived from *Synechococcus* PCC7942) for the overexpression of phosphoribulokinase (PrkA) under the control of P_BAD._	Parikh et al., 2006
rbcLS-pET30a(+)	Recombinant plasmid carries rbcLS gene (originated from *Synechococcus* PCC6301) for the overexpression of engineered RuBisCO under the control of P_T7_	Parikh et al., 2006
pLOI295	Recombinant plasmid carries *pdc* and *adhB* genes (derived from *Zymomonas mobilis*) for overexpression of Pdc-based carbon tap valve under the control of P_lac_	Hespell et al., 1996
pET30b *AfcbbLS*	Recombinant plasmid carries *cbbLS* gene (originated from *A. ferrooxidans* ATCC 23270) for overexpression of RuBisCO under the control of P_7._	Tsai et al., 2015
pAfcbbM	Recombinant plasmid carries *cbbM* gene (AFE_2155) for overexpression of RuBisCO under the control of P_7_.	This study
pAfcbbQ1	The RuBisCO activase gene cbbQ1 (originated from *A. ferrooxidans* ATCC 23270) fused with the His_6_-ubiquitin fragment at the native N termini, was amplified from pHueAfcbbQ1 and cloned into the vector pCDFDuet-1 with *Nco*I/*Hind*III sites.	This study
pAfcbbQ2	The RuBisCO activase gene cbbQ2 (originated from *A. ferrooxidans* ATCC 23270) fused with the His_6_-ubiquitin fragment at the native N termini, was amplified from pHueAfcbbQ2 and cloned into the vector pCDFDuet-1 with *NcoI*/*HindIII* sites.	This study
pAfRca1	The gene *cbbO1* (AFE_3054) was amplified from pBAD33*UbAfcbbO1* and cloned into pAfcbbQ1 vector with *AsisI*-*XhoI*	This study
pAfRca2	The gene *cbbO2* (AFE_2157) was amplified from pBAD33*UbAfcbbO2* and cloned into pAfcbbQ2 with *AsisI*-*XhoI*	This study

### Construction of Recombinant Plasmids

The RuBisCO genes *cbbM* (AFE_2155) were amplified from pHueAfcbbM (Tsai et al., 2015) and cloned between the *NcoI*-*XbaI* sites of the vector *rbcLS*-pET30a(+) to yield pAfcbbM. The Rca genes *cbbQ1* (AFE_3053) and *cbbQ2* (AFE_2156) were amplified from pHueAfcbbQ1 and pHueAfcbbQ2, respectively, (Tsai et al., 2015) and cloned between the *NcoI*/*HindIII* sites of the vector pCDFDuet. This resulted in plasmids pAfcbbQ1 and pAfcbbQ2, respectively. Then, the *cbbO1* (AFE_3054) and *cbbO2* (AFE_2157) were amplified from pBAD33UbAfcbbO1 and pBAD33UbAfcbbO2, respectively, (Tsai et al., 2015) and cloned into the pAfcbbQ1 and pAfcbbQ2 vectors using *Nco*I and *Hin*dIII restriction sites to produce pAfRca1 and pAfRca2. All recombinant plasmids, except pAfRca2, were constructed by sequence and ligation-independent cloning (SLIC) as described below. pAfRca2 was constructed by the traditional digestion ligation cloning method. The primers and their sequences are listed in Supplementary Table S1.

### Sequence and Ligation-Independent Cloning (SLIC)

Six micrograms of vector were digested with restriction enzymes. Isolation and gel purification of the digested vector were carried out using Gene-Spin^TM^ Miniprep Purification Kit (PROTECH, Taiwan) and Gene-Spin ^TM^ 1-4-3 DNA Purification Kit (PROTECH, Taiwan), respectively. Inserts were amplified using *Taq* DNA polymerase. For the iPCR insert, the PCR product was heated to 95°C for 5 min in a water bath for denaturation, followed by slow cooling at room temperature for 1 h for renaturation (Li and Elledge, 2007). One microgram of the vector and one microgram of the inserts were treated separately with 0.5 U of T4 DNA polymerase in T4 buffer (NEB) plus BSA in a 20 μl reaction at room temperature for 30 min (Li and Elledge, 2007). The reaction was stopped by adding 10% (v/v) of 10 mM dCTP and was then kept on ice. The annealing reaction, typically 10 μl, containing an appropriate amount of insert and vector, 1× ligation buffer (NEB), and water, was carried out at 37°C for 30 min, and then the reaction mixture was kept on ice. Finally, *E. coli* cells were transformed using a chemical transformation method (Li and Elledge, 2007).

### Digestion-Ligation Cloning

Six micrograms of vector and insert were prepared by amplification using Q5 DNA polymerase. First, the vector and insert were digested with two different restriction enzymes at 37°C for 2 h. Agarose gel electrophoresis was run with the digested DNA and gel purification was conducted to isolate the DNA using the Gene-Spin ^TM^ 1-4-3 DNA purification Kit. DNA ligation was then carried out with an appropriate amount of vector and insert using T4 DNA ligase (NEB) and 1× T4 DNA ligase buffer in a 20 μl reaction at 4°C overnight. Finally, *E. coli* cells were transformed using a chemical transformation method (Sambrook et al., 1989).

### Culture Media and Conditions

The strains used for fermentation experiments were grown anaerobically at 37°C and then stirred at 200 rpm in fresh 25-mL M9 defined medium containing 111 mM glucose. An anaerobic culture environment was achieved in a sealed serum bottle. A rubber stopper was used to seal the bottle, covered with an aluminum cap, and subjected to heat autoclaving for 20 min.

The initial OD_600_ was adjusted to 0.05. The pH was adjusted to 8 at fermentation times of 0, 8, and 24 h using 2N NaOH. The working concentrations of streptomycin, chloramphenicol, kanamycin, and ampicillin were 50, 34, 50, and 50 μg/mL, respectively. The working concentration of isopropyl-β-D-1-thiogalactopyranoside (IPTG) was 0.02 mM when needed.

### RuBisCO Activity Assay

RuBisCO activity was assayed by adding an appropriate amount of cell-free extract to 700 μL of reaction mixture containing cell lysate, 50 mM HEPES buffer (pH = 8), 1 mM RuBP, 20 mM MgCl_2_, 5 mM DTT, 20 mM NaHCO_3_, 5 mM ATP, 1 mM EDTA, 0.2 mM NADH, 15 U 3-phosphoglycerate kinase, and 6 U of glyceraldehyde 3-phosphate dehydrogenase. Cell-free extract was subjected to carbamylation at 25^⋅^C for 15 min by incubating in a reaction mixture free of RuBP (Tseng et al., 2018). The activity assay was then initiated by adding RuBP, and the consumption of NADH was determined at 25^⋅^C by measuring the UV absorbance at 340 nm. Protein quantification was carried out using the Bradford method (Bio-Rad, United States).

### Analysis of Metabolites

Cell density was measured at 600 nm using a UV-Vis spectrophotometer (Thermo Fisher Scientific, United States). Samples for calculation of extracellular metabolites were collected from the culture media followed by centrifugation for 5 min at 17,000 × *g* to remove cell pellets. The supernatant was filtered through a 0.2-μm PVDF filter before sample injection. Quantification of residual glucose and extracellular formate, acetate, ethanol, lactate, and pyruvate were determined by Thermo ScientificTM DionexTM Ultimate 3000 LC Systems. Separation of the mixture was achieved with the HPLC column Aminex HPX-87H (Bio-Rad, United States) where detection was performed with refractive index (RI, for glucose, acetate, ethanol) and ultraviolet-visible (UV-VIS, for pyruvate, lactate, formate) detectors. The mobile phase was 5-mM H_2_SO_4_. The column temperature was maintained at 45°C, while the flow rate was maintained at 0.6 mL/min. Sample injection (10-μL) was performed using an auto-sampler.

The total CO_2_ production of bacterial cultures was calculated by including gaseous CO_2_, dissolved CO_2_, and hydrated CO_2_ (Zhuang and Li, 2013). Gaseous CO_2_ in the head space of serum bottles was directly measured using an IR-based diffusive spectrometer (Sentry ST303, Taiwan) (Li et al., 2015). Subsequently, dissolved CO_2_ and hydrated CO_2_ were calculated based on the equilibrium constants as described previously (Zhuang and Li, 2013).

### Calculation of Carbon Recovery

The carbon recovery can be calculated using the following equation:

$$\mathrm{Carbon}\mathrm{recovery}\left(\%\right)\;=\frac{\begin{array}{c} {\text{CO}}_{2}+\text{formate}+\text{acetate}\times 2+\text{ethanol}\times 2\\ +\text{pyruvate}\times 3+\text{lactate}\times 3 \end{array}}{\begin{array}{c} \mathrm{glucose}\,\mathrm{consumption}\times 6 \end{array}}$$

where the mols each metabolite are used in the calculation. Note that the biomass is not included.

## Results

### Construction and Expression of RuBisCO and RuBisCO Activase

The maps and verification of pAfcbbM, pAfRca1, and pAfRca2 constructed in this study are shown in Supplementary Figures S1, S2, respectively. The expression of all genes in *E. coli* BL21 (DE3) was confirmed by SDS-PAGE (Supplementary Figure S3). UbcbbM has a theoretical molecular weight of 64.8 kDa, as shown in Supplementary Figure S3A. UbcbbM was found in the soluble fraction. The heterologous co-expression of the UbcbbQ1 and UbcbbO1 in *E. coli* BL21(DE3) was confirmed by SDS-PAGE. UbcbbQ1 and UbcbbO1 have theoretical molecular weights of 93.4 and 38.2 kDa, as shown in Supplementary Figure S3B. UbcbbQ1 was found in the soluble fraction. UbcbbO1 cannot be clearly seen in the soluble fraction, which may be due to its high molecular weight. The heterologous co-expression of UbcbbQ2 and UbcbbO2in *E. coli*BL21(DE3) was confirmed by SDS-PAGE. UbcbbQ2 and UbcbbO2 have theoretical molecular weights of 92.2 and 37.9 kDa, as shown in Supplementary Figure S3C. UbcbbQ2 was found in the soluble fraction. UbcbbO2 cannot be clearly seen in the soluble fraction, which may be due to its high molecular weight.

### Increases in the *in vitro* Activity of RuBisCO in the Presence of RuBisCO Activase (Rca)

Table 2 shows the specific enzyme activity of different forms of RuBisCO in cell extracts of *E. coli* BL21(DE3). Form I RuBisCO (*Synechococcus* PCC6301) exhibited specific activity of 0.3 ± 0.2 μmol mg^–1^ min^–1^, while form I RuBisCO derived from *A. ferrooxidans* ATCC 23270 showed comparable specific activity of 0.5 ± 0.1 μmol mg^–1^ min^–1^. Form II RuBisCO derived from *A. ferrooxidans* ATCC 23270 showed specific activity of 0.5 ± 0.2 μmol mg^–1^ min^–1^.

**TABLE 2:** Specific activities of RuBisCO with RuBisCO activase (Rca) in *E. coli* BL21(DE3) crude lysate^a^.

**Sample^b^**	**Specific Activity of RuBisCO in crude lysate^c^**
	**μmol mg**^–^**^1^ min**^–^**^1^**
rbcLS	0.3 ± 0.2
AfcbbLS	0.5 ± 0.1
AfcbbM	0.5 ± 0.2
rbcLS+ Rca1	1.2 ± 0.3
AfcbbLS+ Rca1	2.2 ± 0.5
AfcbbM+ Rca2	2.6 ± 0.5

*^*a*^ IPTG was supplemented (final concentration = 0.02 mM) for RuBisCO and RuBisCO activase (Rca) expression when OD_600_ of bacterial cultures reached 0.4. RuBisCO and Rca were separately expressed in *E. coli* BL21(DE3). Protein quantification was carried out using the Bradford method.*

*^*b*^ rbcLS represents form I RuBisCO originated from *Synechococcus* PCC6301. AfcbbLS and AfcbbM are form I and form II RuBisCOs originated from *A. ferrooxidans* ATCC 23270. Rca1 and Rca2 are type I and II RuBisCO activases originated from *A. ferrooxidans* ATCC 23270.*

*^*c*^ Errors represent standard deviation with *n* = 3.*

Consistent with previous literature (Tsai et al., 2015), the specific activity of form I RuBisCO (from *A. ferrooxidans*) was increased by adding a second cell lysate containing overexpressed Rca1, and was increased to 2.2 ± 0.5 μmol mg^–1^ min^–1^ (Table 2). Similarly, Rca2 also increased the specific activity of form II RuBisCO (from *A. ferrooxidans*) to 2.6 ± 0.5 μmol mg^–1^ min^–1^. Surprisingly, form I Rca derived from *A. ferrooxidans* also enhanced the specific activity of form I RuBisCO derived from *Synechococcus* PCC6301 and reached 1.2 ± 0.3 μmol mg^–1^ min^–1^ (Table 2).

### Co-expression of RuBisCO Activase (Rca) Enhances Total Metabolite Yields

**FIGURE 1:**
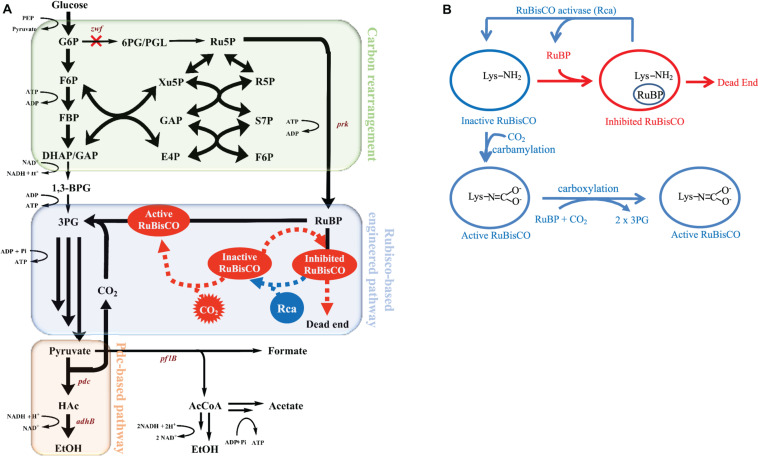
**(A)** The metabolic pathway of mixotrophic *E. coli* with *in situ* CO_2_ recycling and **(B)** schematic of how RuBisCO activase relieves the *in vivo* RuBP inhibition of RuBisCO.

**FIGURE 2:**
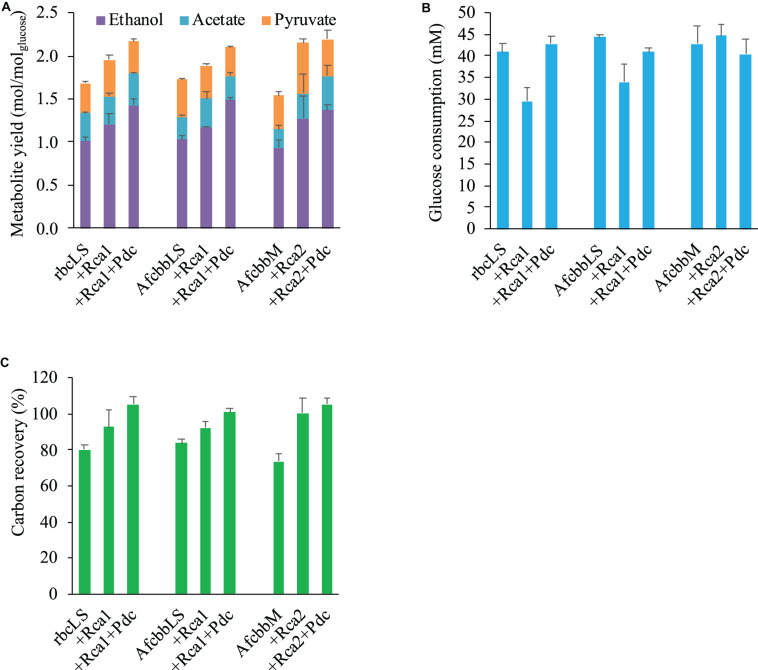
Effects of RuBisCO activase (Rca) co-expression and introduction of Pdc-based pathway (designated as Pdc) on **(A)** total metabolite yields and **(B)** glucose consumption and **(C)** carbon recovery in *Escherichia coli* during *in situ* CO_2_ recycling. Errors represent standard deviation with *n* = 3. Abbreviation: rbcLS represents form I RuBisCO originated from *Synechococcus* PCC6301; AfcbbLS and AfcbbM are form I and form II RuBisCOs originated from *A. ferrooxidans* ATCC 23270. Rca1 and Rca2 are type I and II RuBisCO activases originated from *A. ferrooxidans* ATCC 23270. Pdc represents the Pdc-based pathway consisting of pyruvate decarboxylase (Pdc) and alcohol dehydrogenase (AdhB).

Figure 1A depicts the metabolism of the RuBisCO-based engineered *E. coli*, while Figure 1B shows the schematic of how Rca relieves the *in vivo* RuBP inhibition of RuBisCO. The results shown in Figure 2A for rbcLS, AfcbbLS, and AfcbbM indicate total metabolite yields in the range of 1.5 to 1.7 mol/molglucose, which are not significantly different than observed yields in previous study (Tseng et al., 2018). When Rca was co-expressed with the RuBisCO-based engineered pathway that was equipped with a matching form of RuBisCO, the total metabolite yields of the strains with form I RuBisCO and Rca were found to be close to 2 (2.0 ± 0.1 mol_(ethanol__+__acetate__+__pyruvate)_/mol_glucose_ for rbcLS and 1.9 ± 0.1 mol_(ethanol__+__acetate__+__pyruvate)_/mol_glucose_ for AfcbbLS). A strain with form II RuBisCO with Rca2 exceeded 2 and reached 2.2 ± 0.2 mol_(ethanol__+__acetate__+__pyruvate)_/mol_glucose_ (*p*-values are less than 0.05 for three pairs regarding the Rca co-expression). As shown in Figure 2B, the co-expression of Rca1 decreased glucose consumption from 41 and 44 mM down to 29 and 34 mM for rbcLS and AfcbbLS, respectively. In contrast, the co-expression of Rca2 does not affect glucose consumption (Figure 2B). Figure 2C indicates that the co-expression of Rca has the effect of cascading the carbon flow to the conventional metabolites so that the carbon recovery significantly increased from 80, 84, and 73% to 93, 92, and 100 % for rbcLS, AfcbbLS, and AfcbbM, respectively.

### Introduction of the Pdc-Based Pathway Elevates Both the Performance of the *in situ* CO_2_ Recycling and Glucose Consumption of the *E. coli* Strain Harboring Form I RuBisCO

**FIGURE 3:**
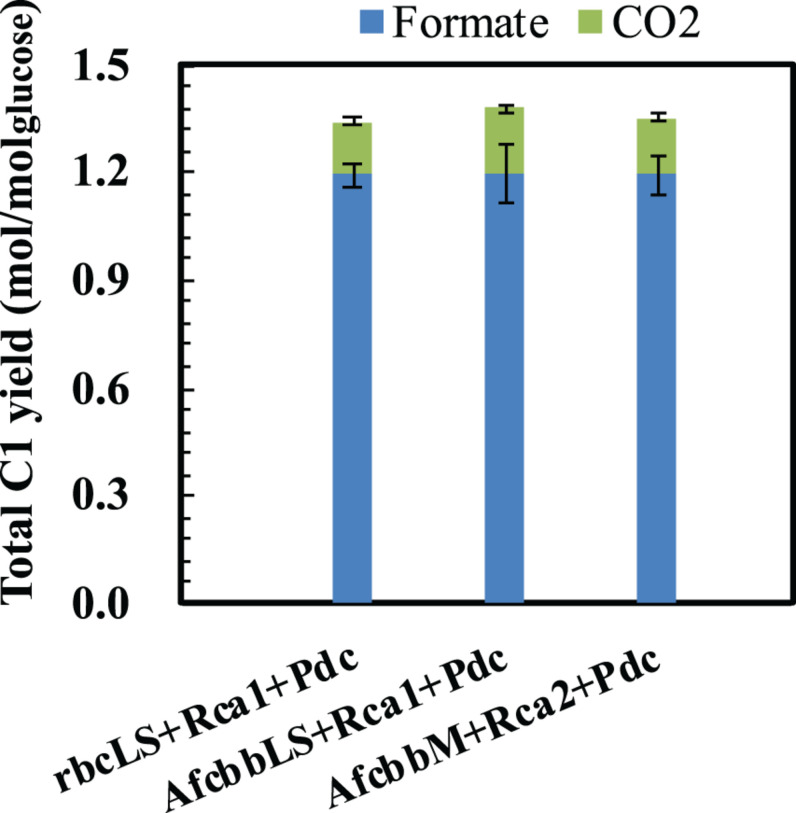
Total C-1 yields of *E. coli* harboring RuBisCO/Rca pair. Errors represent standard deviation with *n* = 3. Abbreviation: rbcLS represents form I RuBisCO originated from *Synechococcus* PCC6301; AfcbbLS and AfcbbM are form I and form II RuBisCOs originated from *A. ferrooxidans* ATCC 23270. Rca1 and Rca2 are type I and II RuBisCO activases originated from *A. ferrooxidans* ATCC 23270. Pdc represents the Pdc-based pathway consisting of pyruvate decarboxylase (Pdc) and alcohol dehydrogenase (AdhB).

In our previous study (Tseng et al., 2018), the Pdc-based pathway (ethanol production pathway) consisting of pyruvate carboxylase (Pdc) and alcohol dehydrogenase (AdhB) (Hespell et al., 1996), (referred to as Pdc in this study) was found to effectively increase glucose consumption by creating ATP demand. A significant result of the Tseng et al. (2018) study was that glucose was diverted to the RuBisCO-based engineered pathway during elevated glucose consumption. Therefore, the performance of *in situ* CO_2_ recycling was accordingly enhanced (Tseng et al., 2018). In this study, Pdc was introduced in the A+Rca system. Figure 2A shows that the introduction of Pdc substantially improves the performance of *in situ* CO_2_ recycling in RuBisCO-based *E. coli* harboring form I RuBisCO. The total metabolite yields of rbcLS+Rca1+Pdc and AfcbbLS+Rca1+Pdc were 2.2 ± 0.1 and 2.1 ± 0.1 mol_(ethanol__+__acetate__+__pyruvate)_/mol_glucose_, which exceeded the conventional fermentation yield of 2 mol_(ethanol__+__acetate__+__pyruvate)_/mol_glucose_. The increases in total metabolite yields illustrated in Figure 2A were primarily due to increased ethanol yields. The high total C-2 (ethanol+acetate) yield of 1.8 mol_(ethanol__+__acetate)_/mol_glucose_ was accompanied by a low total C-1 yield of 1.4 mol_(formate__+__CO2)_/mol_glucose_ (Figure 3), where the C-2/C-1 ratio theoretically should be 1. In addition to increasing the total metabolite yields for the two cases with form I RuBisCO, the introduction of the Pdc-based pathway also recovered the glucose consumption back to 40 mM (Figure 2B) and increased the carbon recovery (Figure 2C) for the two cases with form I RuBisCO. When Rca was co-expressed with the RuBisCO-based engineered pathway that was equipped with a matching form of RuBisCO, the total metabolite yields of the strains with form I RuBisCO and Rca were found to be close to 2 (2.0 ± 0.1 mol_(ethanol__+__acetate__+__pyruvate)_/mol_glucose_ for rbcLS and 1.9 ± 0.1 mol_(ethanol__+__acetate__+__pyruvate)_/mol_glucose_ for AfcbbLS), while a strain with form II RuBisCO with Rca2 exceeded 2 and reached 2.2 ± 0.2 mol_(ethanol__+__acetate__+__pyruvate)_/mol_glucose_ (*p*-values are less than 0.05 for three pairs regarding the Rca co-expression).

## Discussion

This study demonstrated that both form I and form II RuBisCO can be functionally expressed in *E. coli* (Table 2), which is consistent with previous studies which showed that the functional expression of bacterial Rubisco in *E. coli* is not of concern (Parikh et al., 2006; Tsai et al., 2015; Antonovsky et al., 2016; Tseng et al., 2018). The enzymatic assay of RuBisCO indicates that both form I and form II RuBisCO are subject to RuBP inhibition, but the RuBP inhibition can be relieved in the presence of Rca (Table 2). While the *in vitro* RuBP inhibition of RuBisCO shown here is consistent with a previous study (Tsai et al., 2015), this study further revealed that forms I and II RuBisCO also undergo RuBP inhibition *in vivo* because rbcLS, AfcbbLS, and AfcbbM in Figure 2A exhibit insignificant *in situ* CO_2_ recycling. The *in vivo* RuBP inhibition of form I RuBisCO can be alleviated by the co-expression of form I RuBisCO activase (Rca1). This is supported by the result that rbcLS+Rca1 and AfcbbLS+Rca1 have a significant increase in total fermentation product yield (Figure 2A), resulting from the concentrated carbon flow to the RuBisCO-based engineered pathway. Thus, it is suggested that the direction of the carbon flow to the RuBisCO-based engineered pathway is driven by the high activity of Prk when the catalytic power of RuBisCO is sufficient.

The sugar phosphate, RuBP, is known as a tight binder to RuBisCO owing to its low K_M, RuBP_ (McNevin et al., 2006). Therefore, high levels of intracellular CO_2_ are necessary to compete with RuBP to activate RuBisCO by carbamylation. Our previous study showed that a total fermentation yield of 2.17 was achieved at a CO_2_ yield of 0.8 mol/mol_glucose_. In contrast, this study showed that high fermentation product yields (Figure 2A) can be achieved at low CO_2_ yields below 0.2 mol/mol_glucose_ (Figure 3), supporting the remodel of inhibited RuBisCO by Rca.

The effectiveness of Rca co-expression for enhancing *in situ* CO_2_ recycling reflects the substantial issue of *in vivo* RuBisCO inhibition. The *in vivo* RuBisCO inhibition can be overcome by introducing the Pdc-based ethanol biosynthesis pathway (Pdc) so that a significant amount of CO_2_ can be produced to compete with RuBP for activating RuBisCO (Tseng et al., 2018). The *in vivo* RuBisCO inhibition can also be solved by Rca co-expression, as shown in this study. The introduction of Pdc in *E. coli* strains harboring form I RuBisCO provides compensation (Figure 2A), indicating that *in vivo* inhibition of form I RuBisCO is more complex and requires CO_2_ production from the function of Pdc. In fact, the failure of formate dehydrogenase (FDH) co-expression to elevate the performance of *in situ* CO_2_ recycling may simply be because CO_2_ production is too low due to poor FDH activity (Tseng et al., 2018).

It should be noted that the activity of Pdc in the presence of Rca was significantly reduced as acetate and formate were significantly produced (Figure 2A). This could be attributed to the strong ATP demand created by Rca which therefore stimulates the downstream carbon flux to the native *pflB*-mediated pathway to produce acetate and formate, where acetate production is accompanied by ATP production (Schäfer et al., 1993). The trimmed activity of Pdc in the presence of Rca also led to a lower glucose consumption (approximately 40 mM, Figure 2B) compared to that (64 mM) without Rca (Tseng et al., 2018). Note that the extent to which RuBisCO is inhibited in *E. coli* could not be determined based on the activity and fermentation data.

The functional expression of Rca1 can be supported by low glucose consumption of rbcLS+Rca1 and AfcbbLS+Rca1 (Figure 2B). This can be attributed to a high ATP consumption by the Rca1 function, which negatively affects the bacterial growth (data not shown). In contrast, *in vivo* RuBP inhibition of form II RuBisCO can also be addressed with by the co-expression of form II RuBisCO activase (Rca2, Figure 2A). However, AfcbbM+Rca2 has a comparable glucose consumption (Figure 2B) and bacterial growth (data not shown) compared with those in the absence of Rca2. This indicates that reducing the *in vivo* RuBP inhibition of form II RuBisCO may be easier without a significant amount of ATP input. The difference in the ATP demand between forms I and II Rca may be attributed to the plausible role of the small subunit of RuBisCO. Large and small subunits of RuBisCO co-evolve to adapt to environmental changes. Large subunits play a role mainly in catalysis, while small subunits influence not only catalytic efficiency, but also CO_2_ specificity and the assembly and stability of the L_8_S_8_ holoenzyme. Small subunits provide RuBisCO more flexibility to adapt to environmental changes. For example, RuBisCO requires a repeated activation process by Rca to maintain its activity. Although the repeated activation process lowers the catalytic performance of RuBisCO, this makes Rca a good regulator to control RuBisCO activity under certain environmental conditions, such as stromal ATP/ADP ratio (Zhang and Portis, 1999). This study concurs with the above argument that the *in vivo* activity of form I RuBisCO in *E. coli* depends heavily on Rca compared to that of form II RuBisCO. The advantage of using form II RuBisCO for engineering applications is that the *in vivo* RuBP inhibition can be recovered at a low ATP demand.

In past research, it has been demonstrated that the interaction between Rca and RuBisCO comes from the same kind of bacteria (Tsai et al., 2015). In this study, we introduced form I RuBisCO from *Synechococcus* PCC6301 and form I Rca from *A. ferrooxidans* to *E. coli* MZLF. Interestingly, although RuBisCO is from a different species, Rca showed an ability to increase RuBisCO activity, both *in vitro* (Table 2) and *in vivo* (Figure 2). Either Asp82 or HK/R motif at the C-terminus of RuBisCO large subunit has been shown to be essential for *in vitro* activation of the ATPase activity of CbbQ (Tsai et al., 2015). While rbcLS lacks the Asp82 and HK/R motif, the *in vivo* effectiveness of Rca1 in regulating the activity of rbcLS was demonstrated in this study. *In vivo* interactions involving CbbO, CbbQ, and RuBisCO without the HK/R motif could be further investigated.

In summary, both form I and form II RuBisCO undergo *in vivo* RuBP inhibition. On top of the intrinsic low k_cat_ of RuBisCO, *in vivo* RuBP inhibition of RuBisCO has been shown to make apparent k_cat_ low and thus becoming a bottleneck for *in situ* CO_2_ recycling in *E. coli*. The co-expression of Rca can relieve this *in vivo* RuBP inhibition. Furthermore, it is suggested that the *in vivo* RuBP inhibition of form II RuBisCO is easier to address than that of form I RuBisCO. When the catalytic power of RuBisCO is secured, the high activity of Prk plays an important role in directing carbon flow to the RuBisCO-based engineered pathway and produces high fermentation yields of 2.1–2.3 mol_(ethanol__+__acetate__+__pyruvate)_/mol_glucose_.

## Data Availability Statement

The raw data supporting the conclusions of this article will be made available by the authors, without undue reservation.

## Author Contributions

J-JP designed research, carried out experiments, and analyzed data. J-SS analyzed data and wrote the manuscript. S-YL conceived and designed research, analyzed data, and wrote the manuscript. All authors read and approved the final manuscript.

## Conflict of Interest

The authors declare that the research was conducted in the absence of any commercial or financial relationships that could be construed as a potential conflict of interest.

## Abbreviations

RuBisCO: Ribulose-1,5-bisphosphate carboxylase/oxygenase
RuBP: ribulose-1,5-bisphosphate
Rca: RuBisCO activases
CBB cycle: Calvin-Benson-Bassham (CBB) cycle.

## References

[B1] Antonovsky N. Gleizer S. Noor E. Zohar Y. Herz E. Barenholz U. (2016). Sugar Synthesis from CO2 in *Escherichia coli*. *Cell* 166 115–125. 27345370 10.1016/j.cell.2016.05.064 PMC4930481

[B2] Ashida H. Saito Y. Nakano T. Tandeau De Marsac N. Sekowska A. Danchin A. (2008). RuBisCO-like proteins as the enolase enzyme in the methionine salvage pathway: functional and evolutionary relationships between RuBisCO-like proteins and photosynthetic RuBisCO. *J. Exp. Bot.* 59 1543–1554. 10.1093/jxb/ern104 18403380

[B3] Badger M. R. Bek E. B. (2008). Multiple Rubisco forms in proteobacteria: their functional significance in relation to CO2 acquisition by the CBB cycle. *J. Exp. Boot.* 59 1525–1541. 10.1093/jxb/erm297 18245799

[B4] François J. M. Lachaux C. Morin N. (2020). Synthetic biology applied to carbon conservative and carbon dioxide recycling pathways. *Front. Bioeng. Biotechnol.* 7:446. 10.3389/fbioe.2019.00446 31998710 PMC6966089

[B5] Gassler T. Sauer M. Gasser B. Egermeier M. Troyer C. Causon T. (2019). The industrial yeast Pichia pastoris is converted from a heterotroph into an autotroph capable of growth on CO2. *Nat. Biotechnol.* 38 210–216. 10.1038/s41587-019-0363-0 31844294 PMC7008030

[B6] Hespell R. B. Wyckoff H. Dien B. S. Bothast R. J. (1996). Stabilization of pet operon plasmids and ethanol production in *Escherichia coli* strains lacking lactate dehydrogenase and pyruvate formate-lyase activities. *Appl. Environ. Microbiol.* 62 4594–4597. 10.1128/aem.62.12.4594-4597.1996 8953729 PMC168284

[B7] Li M. Z. Elledge S. J. (2007). Harnessing homologous recombination in vitro to generate recombinant DNA via SLIC. *Nat. Methods* 4 251–256. 10.1038/nmeth1010 17293868

[B8] Li Y.-H. Ou-Yang F.-Y. Yang C.-H. Li S.-Y. (2015). The coupling of glycolysis and the Rubisco-based pathway through the non-oxidative pentose phosphate pathway to achieve low carbon dioxide emission fermentation. *Bioresour. Technol.* 187 189–197. 10.1016/j.biortech.2015.03.090 25846189

[B9] McNevin D. Von Caemmerer S. Farquhar G. (2006). Determining RuBisCO activation kinetics and other rate and equilibrium constants by simultaneous multiple non-linear regression of a kinetic model. *J. Exp. Bot.* 57 3883–3900. 10.1093/jxb/erl156 17046981

[B10] Mueller-Cajar O. (2017). The diverse AAA+ Machines that repair inhibited rubisco active sites. *Front. Mol. Biosci.* 4:31. 10.3389/fmolb.2017.00031 28580359 PMC5437159

[B11] Nielsen C. J. Herrmann H. Weller C. (2012). Atmospheric chemistry and environmental impact of the use of amines in carbon capture and storage (CCS). *Chem. Soc. Rev.* 41 6684–6704. 10.1039/c2cs35059a 22729147

[B12] Parikh M. R. Greene D. N. Woods K. K. Matsumura I. (2006). Directed evolution of RuBisCO hypermorphs through genetic selection in engineered *E. coli*. *Protein Eng. Des. Sel.* 19 113–119. 10.1093/protein/gzj010 16423843 PMC2012944

[B13] Parry M. A. J. Keys A. J. Madgwick P. J. Carmo-Silva A. E. Andralojc P. J. (2008). Rubisco regulation: a role for inhibitors. *J. Exp. Bot.* 59 1569–1580. 10.1093/jxb/ern084 18436543

[B14] Salvucci M. E. Portis A. R. Ogren W. L. J. P. R. (1985). A soluble chloroplast protein catalyzes ribulosebisphosphate carboxylase/oxygenase activation in vivo. *Photosynth. Res.* 7 193–201. 10.1007/bf00037012 24443088

[B15] Sambrook J. Fritsch E. F. Maniatis T. (1989). *Molecular Cloning: A Laboratory Manual.* Cold Spring Harbor, NJ: Cold spring harbor laboratory press.

[B16] Schäfer T. Selig M. Schönheit P. (1993). Acetyl-CoA synthetase (ADP forming) in archaea, a novel enzyme involved in acetate formation and ATP synthesis. *Arch. Microbiol.* 159 72–83. 10.1007/bf00244267

[B17] Somerville C. Portis A. R. Ogren W. L. (1982). A mutant of *Arabidopsis thaliana* which lacks activation of RuBP carboxylase in vivo. *Plant Physiol.* 70 381–387. 10.1104/pp.70.2.381 16662500 PMC1067154

[B18] Tabita F. R. Satagopan S. Hanson T. E. Kreel N. E. Scott S. S. (2008). Distinct form I, II, III, and IV Rubisco proteins from the three kingdoms of life provide clues about Rubisco evolution and structure/function relationships. *J. Exp. Bot.* 59 1515–1524. 10.1093/jxb/erm361 18281717

[B19] Tsai Y.-C. C. Lapina M. C. Bhushan S. Mueller-Cajar O. (2015). Identification and characterization of multiple rubisco activases in chemoautotrophic bacteria. *Nat. Commun.* 6:8883. 10.1038/ncomms9883 PMC4660213 26567524

[B20] Tsai Y.-C. C. Ye F. Liew L. Liu D. Bhushan S. Gao Y.-G. (2020). Insights into the mechanism and regulation of the CbbQO-type Rubisco activase, a MoxR AAA+ ATPase. *Proc. Natl. Acad. Sci. U.S.A.* 117 381–387. 10.1073/pnas.1911123117 31848241 PMC6955311

[B21] Tseng I. T. Chen Y.-L. Chen C.-H. Shen Z.-X. Yang C.-H. Li S.-Y. (2018). Exceeding the theoretical fermentation yield in mixotrophic Rubisco-based engineered *Escherichia coli*. *Metab. Eng.* 47 445–452. 10.1016/j.ymben.2018.04.018 29704653

[B22] Yang C.-H. Liu E.-J. Chen Y.-L. Ou-Yang F.-Y. Li S.-Y. (2016). The comprehensive profile of fermentation products during in situ CO2 recycling by Rubisco-based engineered *Escherichia coli*. *Microb. Cell Fact.* 15 1–10. 27485110 10.1186/s12934-016-0530-7 PMC4971712

[B23] Zhang N. Portis A. R. (1999). Mechanism of light regulation of Rubisco: a specific role for the larger Rubisco activase isoform involving reductive activation by thioredoxin-f. *Proc. Natl. Acad. Sci. U.S.A.* 96 9438–9443. 10.1073/pnas.96.16.9438 10430961 PMC17801

[B24] Zhuang Z.-Y. Li S.-Y. (2013). Rubisco-based engineered *Escherichia coli* for in situ carbon dioxide recycling. *Bioresour. Technol.* 150 79–88. 10.1016/j.biortech.2013.09.116 24152790

