# Supplementary material for: The Catalytic Role of RuBisCO for in situ CO2 Recycling in Escherichia coli
Source: Front Bioeng Biotechnol. 2020 Nov 30;8:543807. doi: 10.3389/fbioe.2020.543807 (PMC7734965; doi:10.3389/fbioe.2020.543807)
Supplement: Supplementary file 1 [file Data_Sheet_1.PDF]

Supplementary

Supplementary texts

1. Maps and verification of recombinant plasmids pAfcbbM, pAfRca1, and pAfRca2

The map of recombinant plasmids, pAfcbbM, pAfRca1, and pAfRca2 are shown in Fig S1.

The recombinant plasmid pAfcbbM was digested by *Xba*I.The result is shown in Fig. S2A and the correct size of the fragment is 7.0 kb. The recombinant plasmid pAfRca1 was digested by *Hin*dIII-HF.The result is shown in Fig. S2B and the correct size of the fragments are 2.8 and 4.6 kb. The recombinant plasmid pAfRca2 was digested by*Xho*I.The result is shown in Fig. S2C and the correct size of the fragment is 7.3 kb.

Table S1. Primers used in this study.

Primer	Sequence (5'→3')
SLIC-i-F-*AfcbbM*-01	GAGCGGATAACAATTCCCCTCTAG
SLIC-i-R-*AfcbbM*-01	ttcggatccgatatcagccatgCAGCTTCCTTTCGGGCTTTGTTAG
SLIC-F-*AfcbbQ*-01	ctttaataaggagatataccatgCATATGCAGATCTTTGTGAAGACCCTC
SLIC-R-*AfcbbQ*-01	ttaagcattatgcggccgcaagctCAGCTTCCTTTCGGGCTTTGTTAG
SLIC-F-*AfcbbO*-01	ggatatcggccggccacgcgGCCATATGCAGATCTTTGTGAAGAC
SLIC-R-*AfcbbO*-01	ggtttctttaccagactcgaCTCTCATCCGCCAAAACAGC
dlc-F-*AfcbbO2*-01	ATTATTGCGATCGCGCCATATGCAGATCTTTGTGAAGACCC
dlc-R-*AfcbbO2*-01	ATTATTCTCGAGAAAACAGCCAAGCTTCTACCGGG

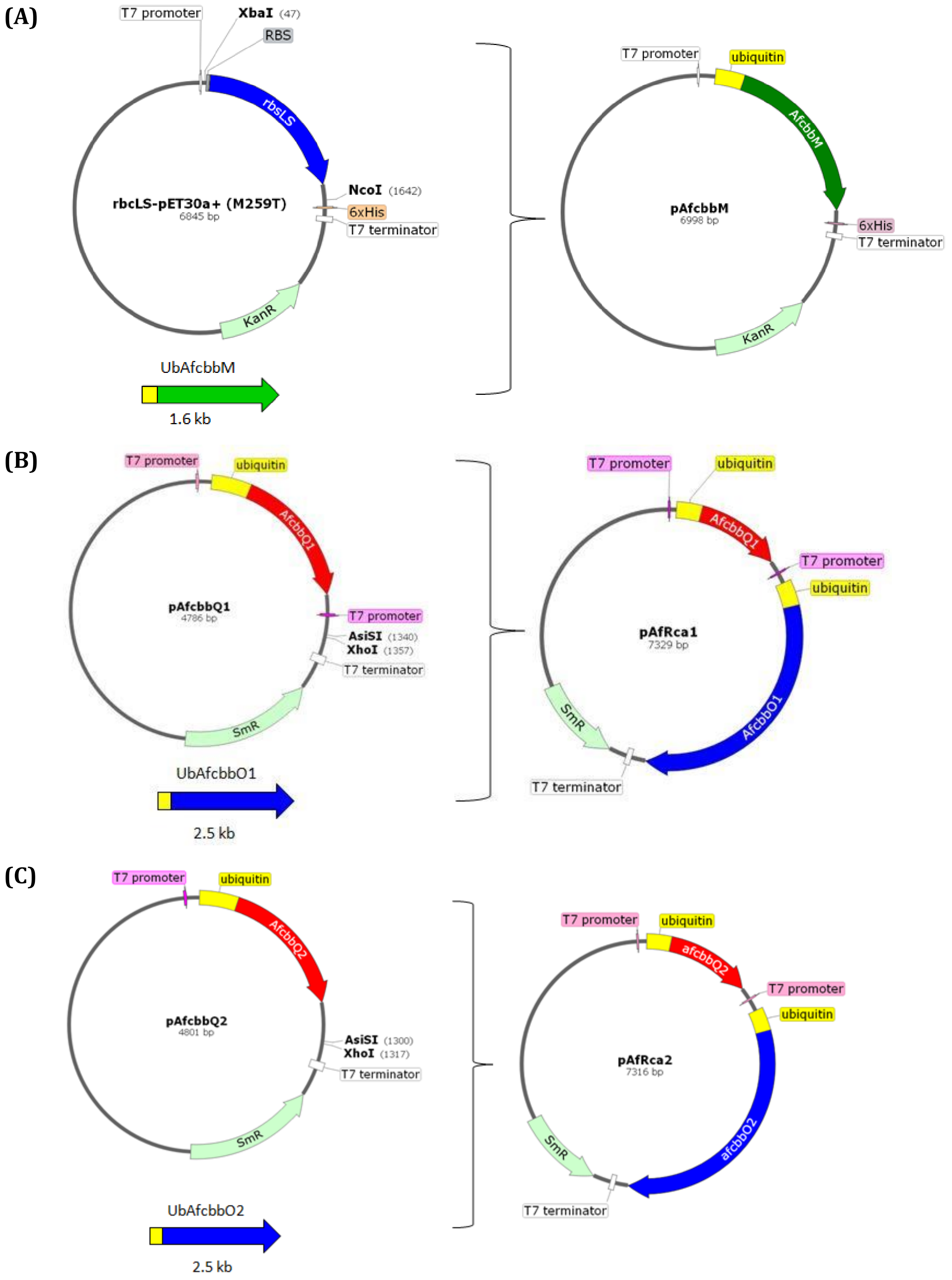

Figure S1. The plasmid maps of the UbAfcbbM-carrying plasmid pAfcbbM, UbAfcbbQ1O1-carrying plasmid pAfRca1, and UbAfcbbQ2O2-carrying plasmid pAfRca2.

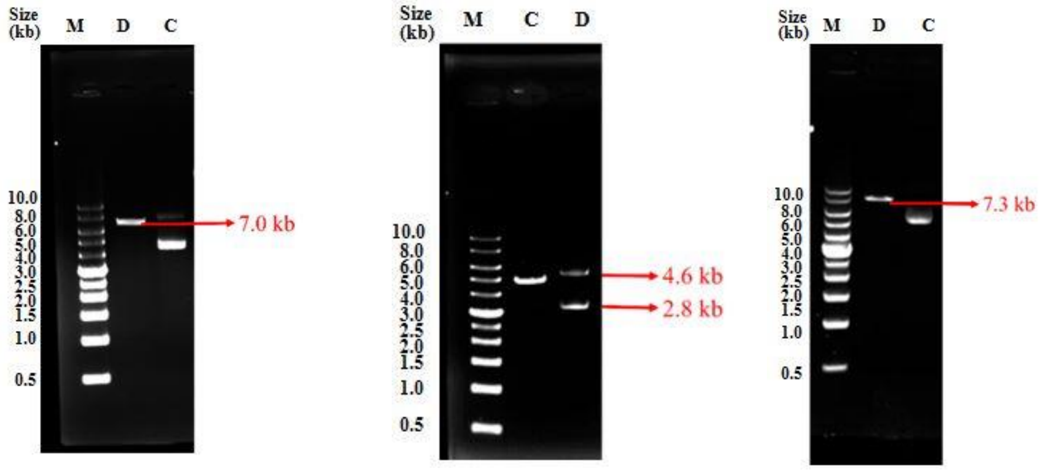

(A) **(B)** **(C)**

Figure S2. The verification of recombinant plasmid enzyme digestion. (A) pAfcbbM was digested by *Xba*I. Lane M: DNA ladder, Lane D: pAfcbbM digested by *Xba*I (arrow shows the 7.9kb band). Lane C: Undigested pAfcbbM. (B) pAfRca1 was digested by *Hin*dIII-HF. Lane M: DNA ladder, Lane C: Undigested pAfRca1. Lane C: pAfRca1 digested by *Hin*dIII-HF (arrows show the 4.6 kb and 2.8 kb bands). (C) pAfRca2 was digested by *Xho*I. Lane M: DNA ladder, Lane D: pAfRca2 digested by *Xho*I (arrow shows the 7.3 kb band). Lane C: Undigested pAfRca2.

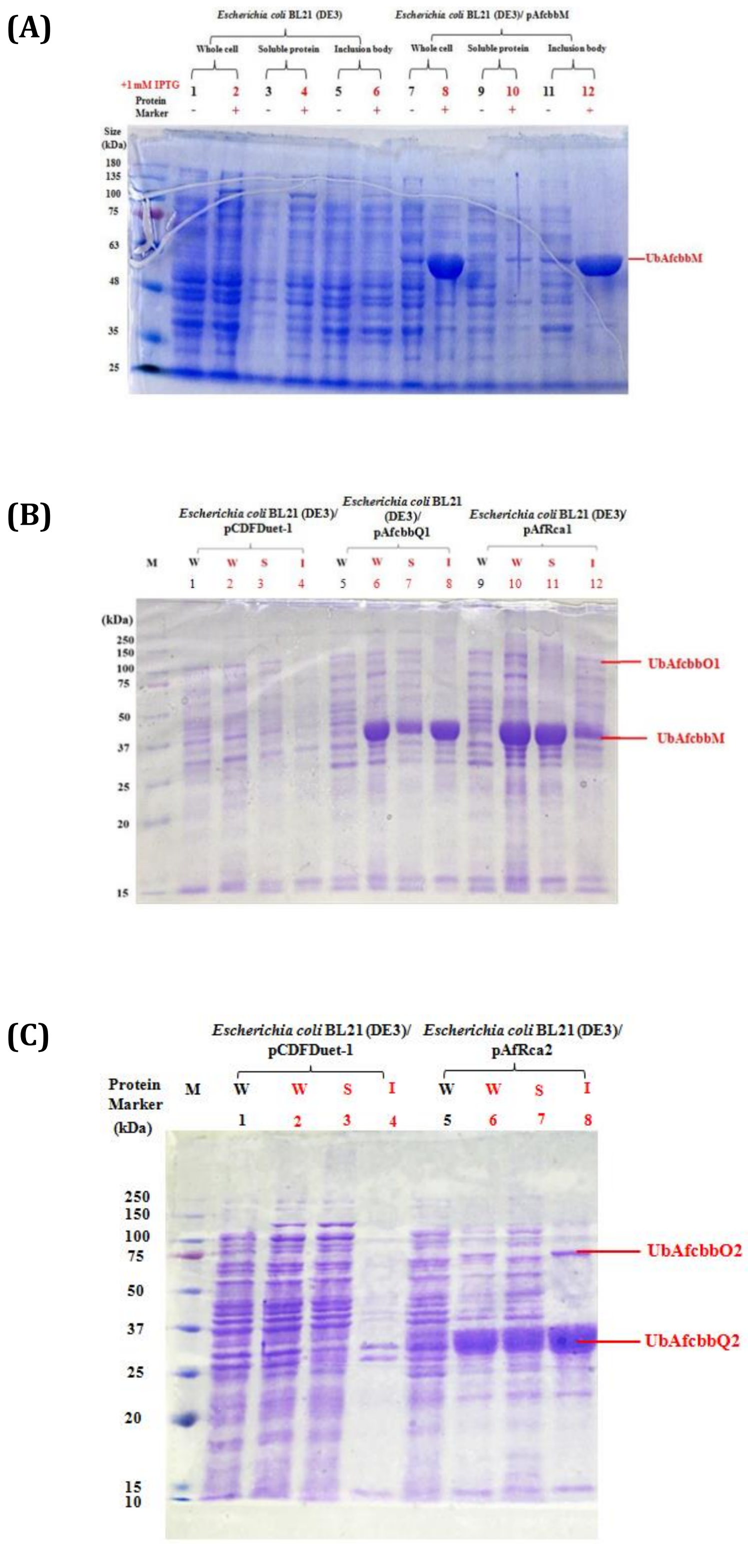

Figure S3. SDS-PAGE analysis of expression for pAfcbbM, pAfRca1, and pAfRca2, (A)Lane M: protein marker; lane 1-6: whole cell, soluble and insoluble fractions of *E. coli* BL21(DE3); Lane 7-12: whole cell, soluble and insoluble fractions of *E. coli* BL21(DE3)/pAfcbbM.(B) Lane M: protein marker; lane 1 and 2: whole cell fraction; lane

3: soluble fraction; lane 4: insoluble fraction of *E. coli* BL21(DE3)/pCDFDuet-1; lane 5 and 6: whole cell fraction; lane 7: soluble fraction; lane 8: insoluble fraction of *E. coli* BL21(DE3)/pAfcbbQ1; lane 9 and 10: whole cell fraction; lane 11: soluble fraction;lane 12 insoluble fraction of *E. coli* BL21(DE3)/pAfRca1. (C) Lane M: protein marker; lane 1 and 2: whole cell fraction; lane 3: soluble fraction;lane 4: insoluble fraction of *E. coli* BL21(DE3)/pCDFDuet-1; lane 5 and 6: whole cell fraction; lane 7: soluble fraction; lane 8: insoluble fraction of *E. coli* BL21(DE3)/pAfRca2. IPTG was added to 1 mM when OD_{600} of each bacterial culture reached around 0.6. UbcbbM, UbcbbQ1, and UbcbbQ2 can be found in the soluble fraction.
